# Integrative assessment of behavioral and cardiorespiratory modulation induced by basil (*Ocimum basilicum* L.) essential oil in tambaqui (*Colossoma macropomum*)

**DOI:** 10.1007/s10695-026-01826-6

**Published:** 2026-11-05

**Authors:** Axell Lins, Luciana Eiró-Quirino, Rômulo Augusto Feio Farias, Rafael dosde ReisSouza, Eduardo Machado da Silva Zamian, João Guilherme Peres, Anezito de Lucas Lopes Corrêa, Beatriz Brilhante de Sousa, Ronaldo de Oliveira Vieira Filho, Melyssa Ferreira Pinheiro Gonzales, Heitor Quaresma Silva, Diogo Macola Felix, Eva Vitória Ferreira Viana, Toniéliton Matheus Gomes Ribeiro, Moisés Hamoy

**Affiliations:** Laboratory of Pharmacology, Toxicology of Natural Products, Federal University of Pará (UFPA), Belém, Brazil

**Keywords:** *Ocimum basilicum*, *Colossoma macropomum*, Fish anesthesia, Essential oil, Electrocardiography, Tambaqui

## Abstract

The use of natural anesthetics, such as essential oils, is increasingly relevant in aquaculture to improve the management of economically important species such as tambaqui (*Colossoma macropomum*). This study investigated the anesthetic potential of basil (*Ocimum basilicum* L.) essential oil (OBEO), primarily composed of estragole (77.71%) and eucalyptol (6.43%), through behavioral, respiratory, and electrocardiographic (ECG) analyses. Tambaqui juveniles (*n* = 90) were exposed to immersion baths containing 76, 85.5, 95, or 104.5 mg L⁻^1^ OBEO. OBEO produced a concentration-dependent behavioral anesthetic state characterized by loss of postural reflex and immobility, with shorter induction latencies and longer recovery times at higher concentrations. Because responses to noxious stimuli were not evaluated, these endpoints demonstrate immobilization appropriate for routine handling rather than a confirmed surgical plane of anesthesia. Sinus bradycardia increased with concentration, reaching a 40.83% reduction in heart rate at 104.5 mg L⁻^1^ relative to the control. RR, PQ, ST, and QRS duration increased at higher concentrations, whereas QRS complex amplitude remained unchanged. Opercular frequency and power also decreased in a concentration-dependent manner. During the 5-min washout in anesthetic-free water, behavioral and most mean cardiorespiratory variables moved toward control values, although recovery was slower and some ECG variables remained different at the highest concentration. Under these short-term experimental conditions, no arrhythmias or persistent gross physiological abnormalities were observed. OBEO therefore shows potential as a reversible immobilizing/anesthetic agent for routine handling of juvenile tambaqui; broader conclusions regarding food-fish safety and practical use require pharmacokinetic, residue, toxicological, and withdrawal-period studies.

## Introduction

Historically, the use of medicinal plants as a source of therapeutic agents has played a fundamental role in the development of new drugs, remaining a relevant strategy in regions of high biodiversity, such as Brazil. Due to their wide range of biological properties including antiseptic, antibiotic, antiviral, and anti-inflammatory activities these compounds have found growing applications in diverse sectors, such as the food and beverage industry, the fragrance sector, alternative medicine, and, with particular emphasis, the pharmaceutical industry (Sattayakhom et al. 2023).

A notable example of a widely used essential oil is *Ocimum basilicum* L., popularly known as basil. Originating from Asia and Africa and a member of the Lamiaceae family, this aromatic plant is of great global relevance, with its essential oil being extensively employed in the food industry (Kamelnia et al. 2023; de Souza et al. 2025).

In this context, anesthetics play a crucial role. These compounds induce a temporary and reversible suppression of the central nervous system, resulting in the loss of sensitivity and the inability to react to external stimuli (Ross and Ross 2008). In aquaculture, the use of anesthetics has proven increasingly relevant for mitigating stress in farming environments, contributing to improved handling and promoting fish welfare (Barbas and Hamoy 2021).

Among the fish species employed in aquaculture, the tambaqui (*Colossoma macropomum*), belonging to the order *Characiformes*, stands out for its economic relevance in Northern Brazil (Costa et al. 2022; Lins et al. 2025). This fish is notable for its ability to respond to various drugs and for its resistance to handling, characteristics that make it a promising model for electrophysiological studies (Barbas et al. 2016).

Electrocardiography (ECG) provides a direct measure of cardiac electrical activity and is increasingly used in fish to characterize baseline cardiovascular physiology, autonomic responses, and electrophysiological effects of anesthetics and candidate cardiotoxic compounds (Cotter and Rodnick 2006; Zhao et al. 2019; Ling et al. 2022; Cantanhêde et al. 2020). In anesthetized fish, interpretation may integrate rhythm and heart rate (HR), beat-to-beat variability, RR interval, atrioventricular conduction, QRS morphology and duration, and ventricular repolarization indices such as the QT interval when wave boundaries can be identified reliably (Cotter and Rodnick 2006; Zhao et al. 2019). ECG has also been used as a complementary tool to evaluate plant-derived anesthetics in live fish, including tambaqui (Barbas et al. 2017; de Araújo et al. 2023; Vilhena et al. 2022). These measurements are particularly useful because different anesthetic agents can produce distinct effects on cardiac rate, conduction, repolarization, and autonomic modulation, emphasizing the need for agent- and species-specific physiological evaluation.

The continuous advancement of aquaculture as one of the main sectors producing animal protein has intensified the adoption of increasingly frequent and technically complex management practices, such as biometrics, transportation, induced reproduction, and sanitary procedures. When carried out without proper control, these interventions can trigger significant physiological and behavioral changes in fish, compromising productive performance, immunocompetence, and animal survival. In this context, the use of anesthetic agents constitutes an essential tool to promote animal welfare during aquaculture management. However, currently employed anesthetics present relevant limitations, including dose-dependent side effects, prolonged recovery times, potential environmental impact, and regulatory restrictions regarding their use in species intended for human consumption (Zahl et al. 2012; Skår et al. 2017). Therefore, the use of natural anesthetics is becoming more widespread.

In parallel, despite the growing number of investigations involving *Ocimum basilicum* L., considerable heterogeneity is observed in the reported results, often associated with variations in the type of extract, extraction conditions, phytochemical composition, and experimental models used. Furthermore, many studies are restricted to the description of biological activities without an in-depth comparative analysis or consistent mechanistic discussion. Given this scenario, the need to more robustly elucidate the sedative effects of *O. basilicum* becomes evident, to ensure its efficacy and safety, as well as to evaluate its potential as a new pharmacological alternative for fish handling and aquaculture.

## Experimental section

### Experimental animals

Specimens of the tambaqui species (*Colossoma macropomum*) (*n* = 90) were housed in four 200 L aquaria at the Experimentation Animal Facility of the Laboratory of Pharmacology and Toxicology of Natural Products at the Federal University of Pará (ICB/UFPA). The system was equipped with a submersible pump for oxygenation and maintained in a temperature-controlled environment (25 to 27 °C) with a 12 h L: 12 h D photoperiod. Feeding was performed twice daily with commercial feed (32% protein). Daily water renewal of 30% of the system volume was performed using water from the same source, along with siphoning procedures to remove uneaten food and feces. During acclimatization (10 days), water quality variables such as water temperature (26.1 °C) and pH (7.63) were monitored. The project was approved by CEUA-UFPA under number 6600260924 (ID 002764).

### Organoleptic properties and chromatographic analysis

The essential oil of *Ocimum basilicum* L. was obtained from Laboratory Harmonie (Registry: 11.938.821/0001–90), extracted by steam distillation, and analyzed by the Department of Chemical Engineering and Food Engineering (EQA) at the Federal University of Santa Catarina. The analysis method used was High-Performance Gas Chromatography on an AGILENT 7820 A Gas Chromatograph under the following conditions: Column: HP-5 30 m × 0.32 mm × 0.25 μm (AGILENT). Temp.: Column: 70 °C (0 min), increasing 3 °C/min to 250 °C. Injector: 250 °C. Split: 1/50. Detector FID: 260 °C. Injection vol.: 1 µL (1% in chloroform).

**Fig. 1:**
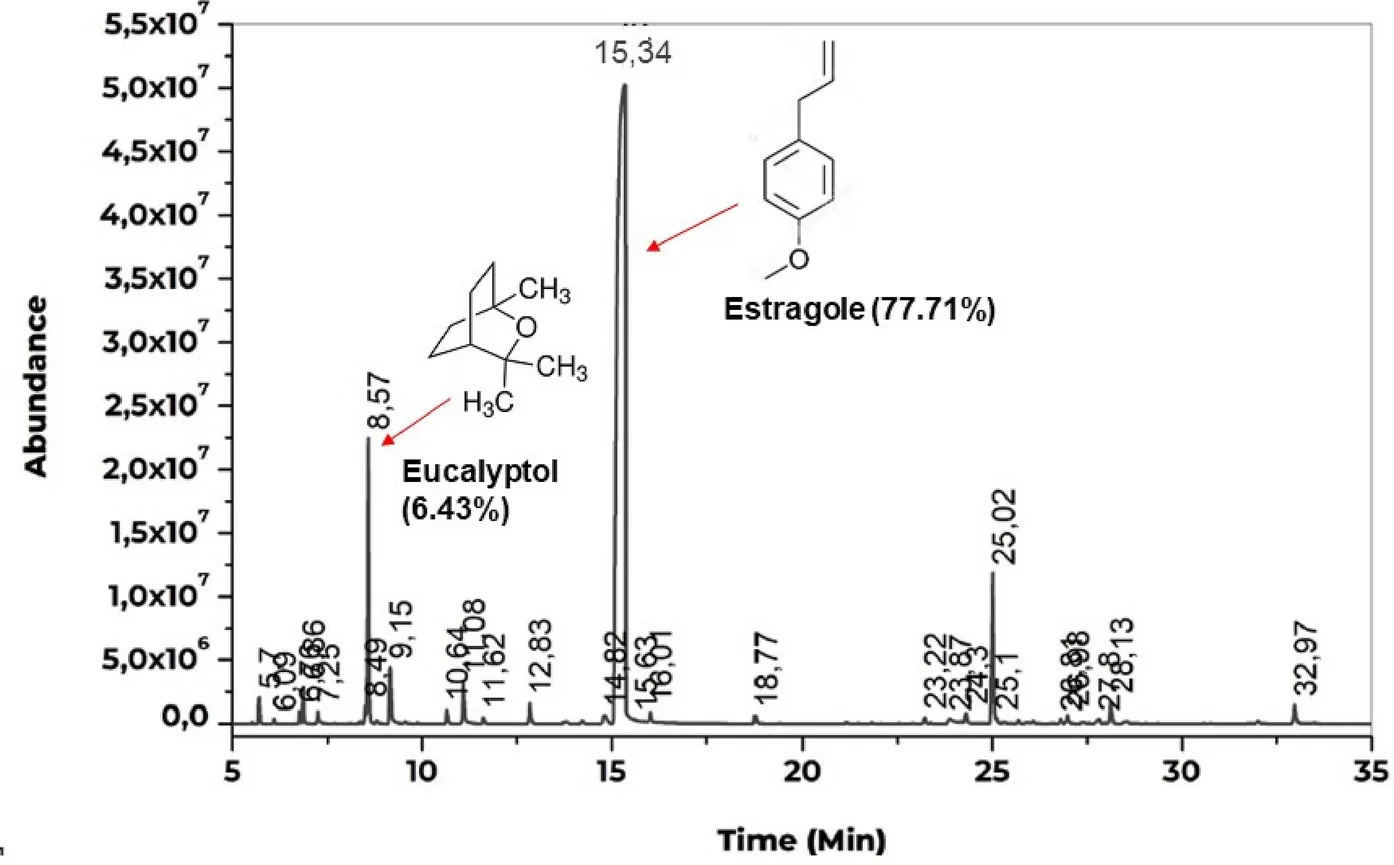
Chromatogram of the OBEO essential oil sample, gas chromatography–mass spectrometry (GC–MS). Department of Chemical Engineering and Food Engineering (EQA) of the Federal University of Santa Catarina, Brazil

The oil presented characteristics such as an herbal aroma and light-yellow color, with a density of 0.950 g/cm^3^. The phytoconstituents composing the oil are described in Table 1, with the major components being Estragole (77.71%) and Eucalyptol (6.43%). It presents a purity grade of 100% and, being an oleaginous substance, it was diluted in 70% alcohol at a ratio of 1 mL of oil to 9 mL of alcohol and stored in a refrigerator at 4 °C for subsequent aliquot withdrawal for the experiment (Fig. 1).

**Table 1:** Phytoconstituents that compose the OBEO

Retention time	Substance identification	%
5.70	α-Pinene	0.45
6.77	Sabinene	0.25
6.87	β-Pinene	0.73
7.25	Myrcene	0.35
8.49	D-Limonene	0.38
8.57	Eucalyptol	6.43
8.80	Cis-β-Ocimene	0.10
9.16	Trans-β-Ocimene	1.49
10.64	Fechone	0.38
11.09	Linalool	1.20
11.62	Fenchol	0.16
12.83	Camphor	0.60
14.22	Terpinen-4 -ol	0.11
14.82	α-Terpineol	0.49
15.34	Estragole	77.71
16.01	Fenchyl acetate	0.37
18.77	Bomyl acetate	0.23
23.22	β-Elemene	0.17
23.88	Methyleugenol	0.41
24.30	Caryophyllene	0.35
25.02	Trans- α- Bregamotene	4.53
26.81	Germacrene D	0.13
27,80	α-Bulnesene	0.21
28.13	γ- Cardinene	0.68
28.54	δ—Cardinene	0.12
32.97	T—Cardinol	0.64
	Minority compounds (< 0.10%)	0.92
	Unidentified compounds	0.40

## Experimental design

### Experiment with Ocimum basilicum L. essential oil

Tambaqui juveniles (26.63 ± 5.1 g) were randomly allocated to the following treatments: (a) control; (b) vehicle (1.1 mL of 70% alcohol per 1 L of aquarium water); (c) OBEO 76 mg L⁻^1^; (d) OBEO 85.5 mg L⁻^1^; (e) OBEO 95 mg L⁻^1^; and (f) OBEO 104.5 mg L⁻^1^. The induction/exposure phase lasted 5 min, followed by a 5-min recovery (washout) period in anesthetic-free water. For each recording, *n* = 9 fish per treatment was used. Each animal participated in only one experimental protocol and was not reused between behavioral and electrophysiological experiments.

#### Experiment 1—analysis of characteristic behaviors of anesthetic induction and recovery

For OBEO treatments (76, 85.5, 95, and 104.5 mg L⁻^1^; *n* = 9/treatment), behavioral induction was quantified as the latency to loss of the postural reflex and to immobility, characterized by lateral recumbency and loss of spontaneous somatic activity. These criteria were used operationally to define the behavioral anesthetic/immobilization state required for routine handling. A surgical plane of anesthesia was not assessed because responses to noxious stimulation and analgesic endpoints were not tested. After the 5-min exposure, fish were transferred to anesthetic-free water, and the latencies to recovery of the postural reflex and spontaneous somatic activity were recorded.

#### Experiment 2—analysis of opercular activity and electrocardiogram recordings during anesthetic induction and recovery

For electrophysiological recordings, the groups were (a) control; (b) vehicle; (c) OBEO 76 mg L⁻^1^; (d) OBEO 85.5 mg L⁻^1^; (e) OBEO 95 mg L⁻^1^; and (f) OBEO 104.5 mg L⁻^1^ (*n* = 9 per group; total = 54 fish).

Each fish was tested individually. ECG and opercular electrodes were positioned before exposure to the assigned treatment bath. Electrode implantation was performed under brief manual restraint and required approximately 1–2 min per fish; after placement, the fish remained for an additional 5 min for accommodation and signal stabilization before the timed exposure began. The fish was then transferred to the assigned control, vehicle, or OBEO bath, and the continuous 5-min induction/exposure recording was started immediately at immersion (*t* = 0) while the fish remained in that solution. At 300 s, OBEO-treated fish were transferred directly to clean, anesthetic-free water with the electrodes maintained in position, and a second continuous 5-min recording was started immediately to characterize washout/recovery. Accordingly, Fig. 3 represents recordings obtained during active treatment exposure, whereas Fig. 4 represents recordings obtained after transfer to anesthetic-free water.

To acquire opercular activity, electrodes were fabricated from 925 silver with a diameter of 0.5 mm and a length of 20 mm. The electrodes were conjugated at 7 mm and insulated with liquid insulation (Quimatic). The electrode placement for recording the opercular beat was the central part of the right opercular opening. During the recordings, frequency (opercular movements per minute) and opercular beat power (mV^2^/Hz) were evaluated.

For cardiac recordings, electrodes were fabricated from 925 silver (0.3 mm diameter, 12 mm length), insulated with liquid insulation (Quimatic), and used in a non-conjugated configuration. The reference electrode was positioned to indicate the cardiac vector (negative pole at the base of the heart and positive pole at the apex) and was fixed in the ventral portion of the left operculum, 0.2 mm before the end of the opercular cavity. The recording electrode was inserted 2.0 mm before the end of the right opercular opening, providing a signal approximating Lead I (D1). During implantation, fish were manually restrained with a damp cloth to minimize stress and injury. The implantation site was locally anesthetized with 2% lidocaine applied on impregnated cotton before insertion of the hook-shaped electrode. After electrode placement, a 5-min accommodation period was allowed before the treatment recording began. Electrodes were connected to a high-impedance amplifier (Grass Technologies, Model P511). The measured ECG variables were heart rate (bpm), QRS complex peak-to-peak amplitude (mV), QRS duration (ms), RR interval (ms), PQ interval (ms), and ST interval (ms). QRS amplitude was defined as the voltage difference between the maximum positive and minimum negative deflections of the QRS complex. RR was measured between consecutive R peaks; PQ from the onset of the P wave to the onset of the QRS complex; QRS duration from the first to the last deflection of the QRS complex; and ST from the end of the S wave to the onset of the T wave. The QT interval was not included because the terminal boundary of the T wave could not be identified with sufficient consistency across all recordings, particularly during higher-concentration exposure, precluding reliable QT/QTc measurement. A representative annotated ECG segment illustrating the measured variables is provided in Fig. 3M.

### Recording and data analysis

Data analysis was performed using a tool built in the Python programming language. The NumPy and SciPy libraries were used for mathematical processing, and the Matplotlib library was used for graph creation. The graphical interface was developed using the PyQt4 library (Lins et al. 2025).

The electrodes were connected to a digital data acquisition system via a high-input-impedance differential amplifier (Grass Technologies, Model P511), adjusted with filtering between 0.3 and 300 Hz and amplification of 2000×, and monitored with an oscilloscope (Protek, Model 6510). Recordings were continuously digitized at a rate of 1 kHz on a computer equipped with a data acquisition board (National Instruments, Austin, TX, USA), stored on a hard drive, and subsequently processed using specialized software (LabVIEW Express).

#### Statistical analysis

After evaluating compliance with the assumptions of normality and homogeneity of variances using the Kolmogorov–Smirnov and Levene tests, respectively, comparisons of mean power values were performed using one-way ANOVA, followed by Tukey’s test. The Bonferroni correction was performed using Python and the statsmodels library. Cohenʼs d effect size values were calculated using Python and the Pingouin library. GraphPad Prism 8 software was used for analyses, and values of *p < 0.05, **p < 0.01, and ***p < 0.001 were considered statistically significant in all cases.

## Results

**Fig. 2:**
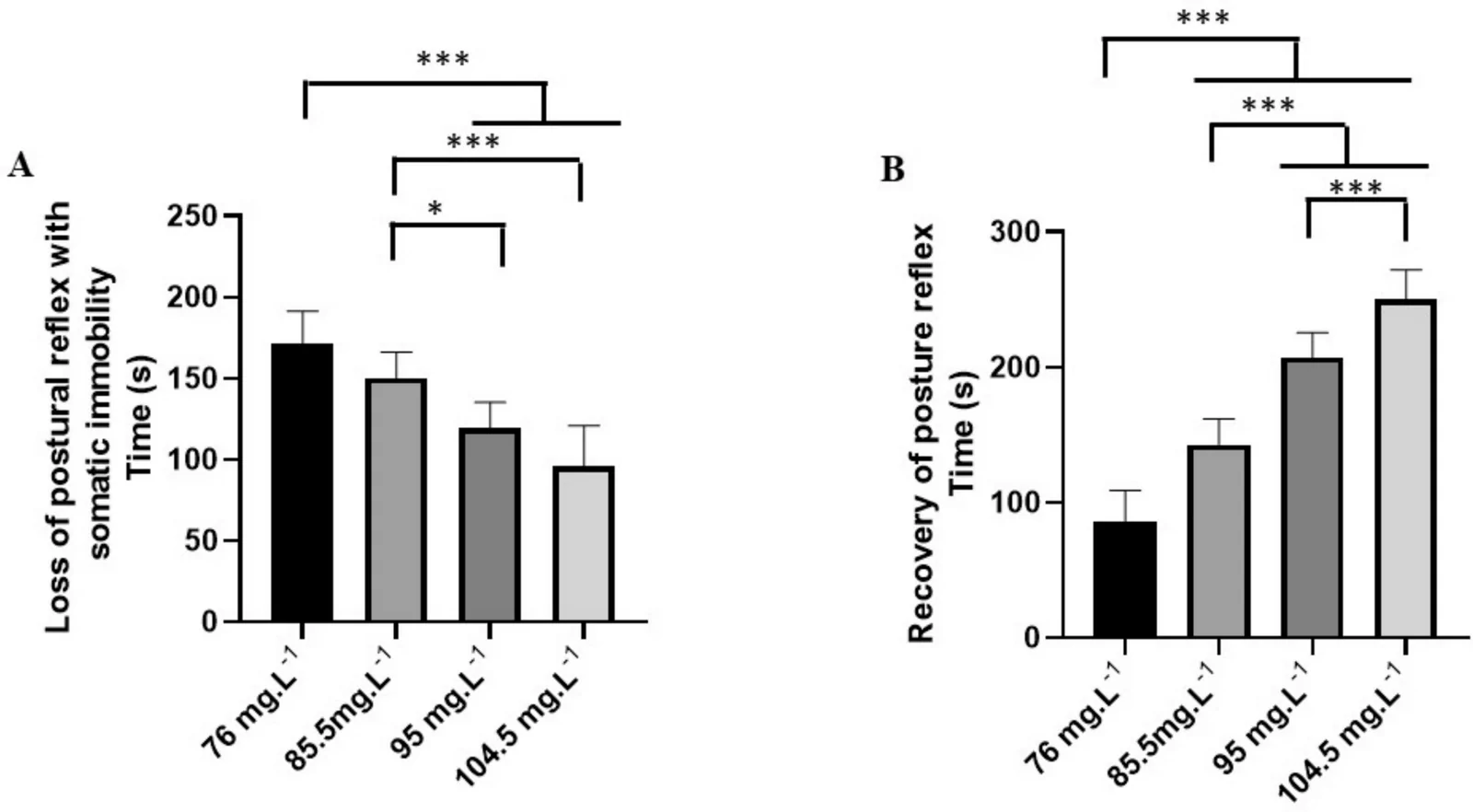
Mean latencies in seconds for loss of postural reflex during immersion baths with different OBEO treatments **A**. Recovery of postural reflex after treatment with different concentrations of OBEO **B**. (ANOVA followed by Tukey’s test; *p < 0.05, **p < 0.01, and ***p < 0.001)

Behavioral analysis showed that OBEO induced loss of the postural reflex and immobility, with shorter induction latencies at higher concentrations. Fish treated with 76 mg L⁻^1^ presented a mean latency to loss of the postural reflex of 171.3 ± 19.99 s, similar to the 85.5 mg L⁻^1^ group (*p* = 0.122). The 95 mg L⁻^1^ group presented a mean latency of 119.9 ± 15.41 s, with no significant difference from the 104.5 mg L⁻^1^ group (*p* = 0.0602) (Fig. 2A). These behavioral endpoints indicate effective immobilization; they should not be interpreted as evidence of a surgical anesthetic plane because noxious-stimulus responses were not evaluated.

The latency to recovery of the postural reflex was 86.44 ± 22.78 s in fish treated with 76 mg L⁻^1^ OBEO and increased with concentration: 142.8 ± 19.08 s at 85.5 mg L⁻^1^, 207.4 ± 18.17 s at 95 mg L⁻^1^, and 250.6 ± 21.64 s at 104.5 mg L⁻^1^. Thus, higher concentrations required progressively more time for behavioral recovery after transfer to anesthetic-free water (Fig. 2B).

**Fig. 3:**
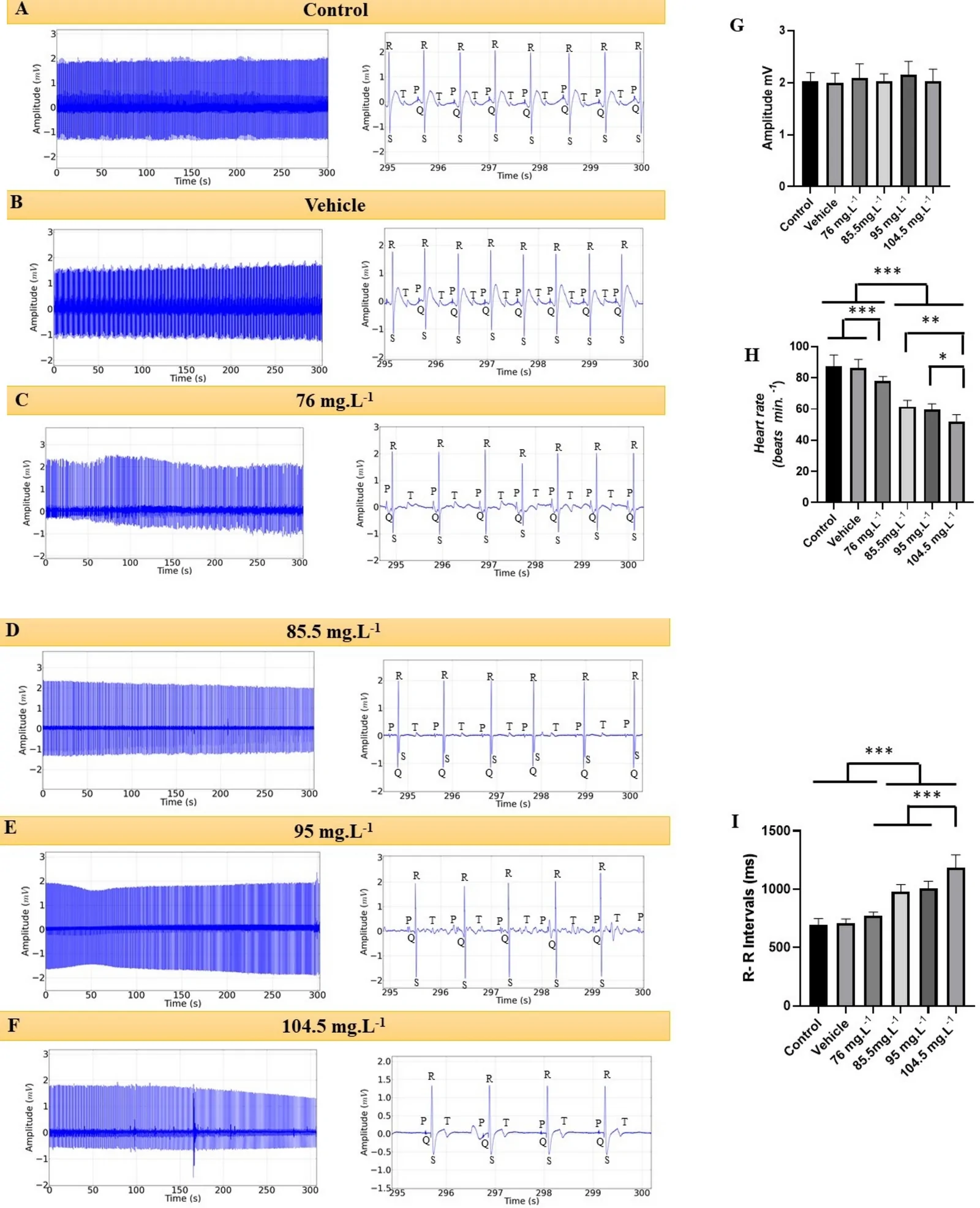

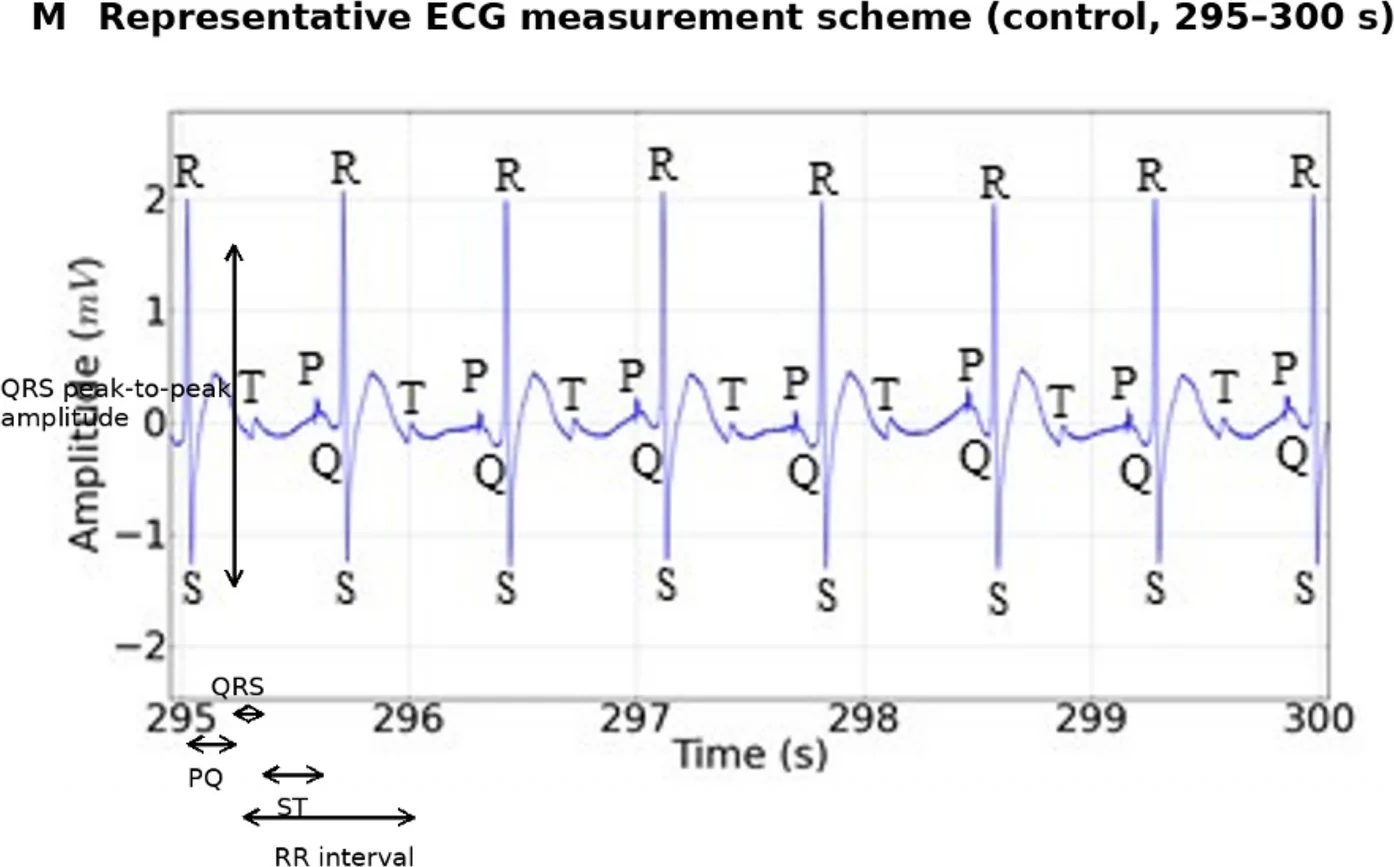
Electrocardiographic recordings demonstrating cardiac activity in juvenile *Colossoma macropomum* (left) and 5-s amplification at the end of the 5-min exposure recording (295–300 s; center) for Control **A**, Vehicle **B**, 76 mg L⁻^1^ **C**, 85.5 mg L⁻^1^ **D**, 95 mg L⁻^1^ **E**, and 104.5 mg L⁻^1^ OBEO **F**. Quantitative analyses: QRS complex peak-to-peak amplitude (mV) **G**, heart rate (bpm) **H**, RR interval (ms) **I**, PQ interval (ms) **J**, QRS duration (ms) **K**, and ST interval (ms) **L**. Panel 3M is an annotated representative control ECG segment illustrating the operational definitions of RR, PQ, QRS duration, ST, and QRS peak-to-peak amplitude. ANOVA followed by Tukey’s test; data are mean ± standard deviation; *p < 0.05, **p < 0.01, and ***p < 0.001; *n* = 9

The normal electrocardiogram of *Colossoma macropomum* presented a sinus rhythm and a mean heart rate of 87.33 ± 7.28 bpm; identified graphoelements included the P wave, the QRS complex, and the T wave (Fig. 3A). Cardiac activity in the control group was like the vehicle group (*p* = 0.999) (Fig. 3B). Based on a 5-s amplification, interval evaluations were performed during OBEO immersion bath treatment (Fig. 3C–F).

Relative to the control heart rate (87.33 ± 7.28 bpm), OBEO produced concentration-dependent sinus bradycardia. Heart rate was 77.78 ± 3.07 bpm at 76 mg L⁻^1^ (10.93% reduction) and 61.56 ± 4.09 bpm at 85.5 mg L⁻^1^ (29.50% reduction); reductions relative to control were 31.79% at 95 mg L⁻^1^ and 40.83% at 104.5 mg L⁻^1^ (Fig. 3C–F, H). The 85.5 and 95 mg L⁻^1^ groups were similar (*p* = 0.952), whereas the 104.5 mg L⁻^1^ group showed the greatest reduction. The vehicle did not differ from control (*p* = 0.999).

The mean QRS complex amplitude for the control group was 2.02 ± 0.174 mV, which was similar to the vehicle group and all groups receiving OBEO treatment (F (5, 48) = 0.681; *p* = 0.639) (Fig. 3G).

The mean RR interval for the control group was 691.0 ± 56.76 ms, which was like the mean RR interval for the vehicle group (*p* = 0.991) and the 76 mg L⁻^1^ group (*p* = 0.099); however, these were lower compared to the other OBEO-treated groups. The group treated with 85.5 mg L⁻^1^ had a mean RR interval of 977.0 ± 62.67 ms, which was similar to the group treated with 95 mg L⁻^1^ (*p* = 0.891). The 104.5 mg L⁻^1^ group (1184 ± 110.2 ms) was higher than the other groups (Fig. 3I).

The mean PQ interval for the control group was 115.9 ± 5.08 ms, similar to the vehicle group (p = 0.999) and the group treated with 76 mg L⁻^1^ (*p* = 0.481). The group treated with 85.5 mg L⁻^1^ (123.3 ± 5.05 ms) was similar to the group treated with 95 mg L⁻^1^ (*p* = 0.999). The group treated with 104.5 mg L⁻^1^ (188.0 ± 11.36 ms) presented a PQ interval higher than the other groups (Fig. 3J).

The mean QRS complex duration for the control group (20.22 ± 2.89 ms) was similar to the vehicle group (*p* = 0.999) and those treated with 76 mg L⁻^1^ (*p* = 0.999), 85.5 mg L⁻^1^ (p = 0.461), and 95 mg L⁻^1^ (*p* = 0.052). However, they were lower than the group treated with 104.5 mg L⁻^1^ (29.00 ± 2.69 ms) (Fig. 3K).

For the control group, the mean ST interval was 290.7 ± 10.15 ms, which was similar to the vehicle group (*p* = 0.999), but lower than the other groups. The group treated with 76 mg L⁻^1^ (310.4 ± 10.27 ms) was lower than the other treated groups. The group treated with 85.5 mg L⁻^1^ (343.3 ± 13.30 ms) was similar to the group treated with 95 mg L⁻^1^ (*p* = 0.943). The group treated with 104.5 mg L⁻^1^ (373.3 ± 18.28 ms) was higher than the other groups (Fig. 3L).

**Fig. 4:**
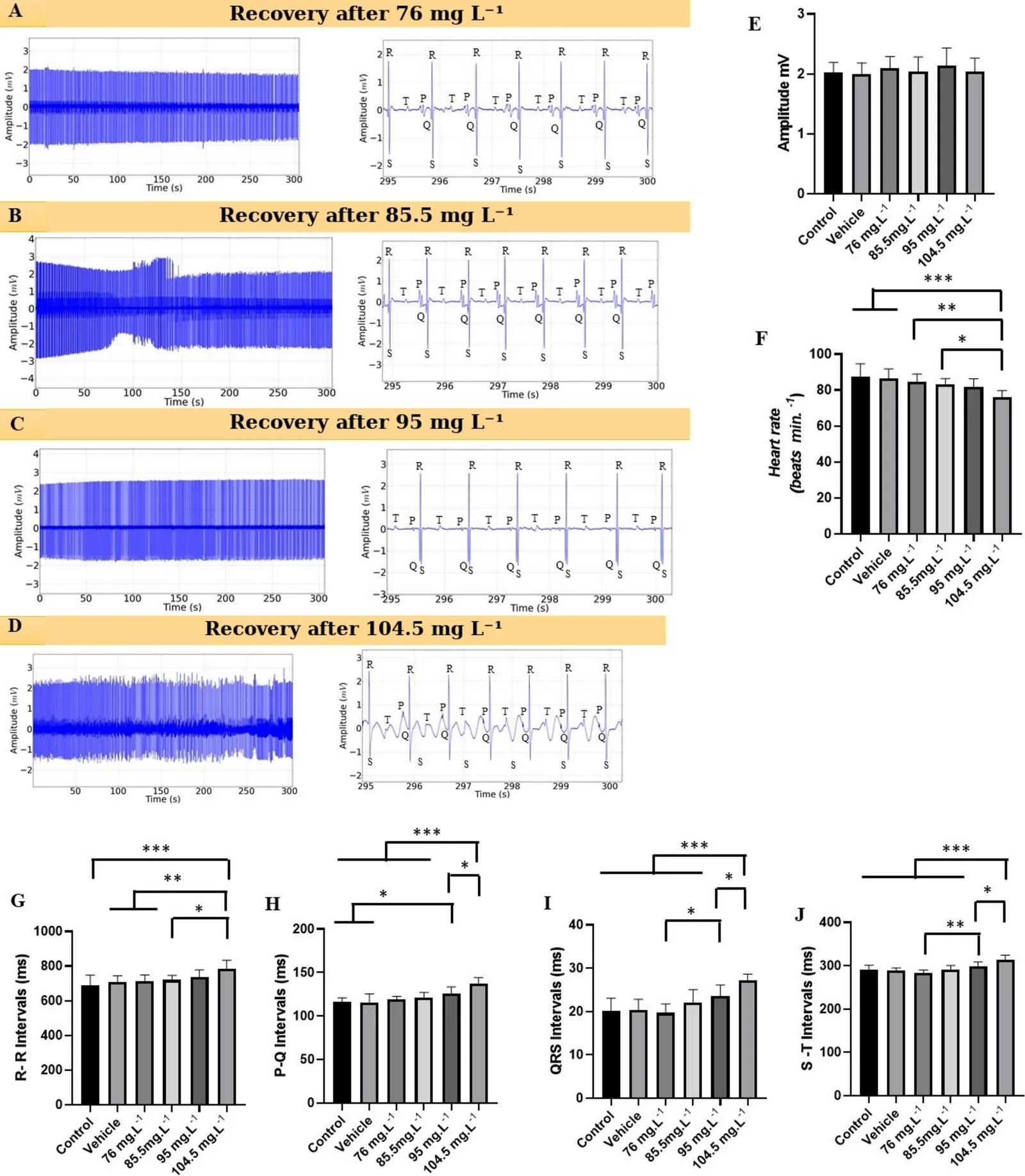
Cardiac assessment during the 5-min recovery/washout period after OBEO exposure in juvenile *Colossoma macropomum*. All traces in panels **A**–**D **were recorded after the fish had been transferred to clean, anesthetic-free water; the concentration shown above each trace identifies the preceding 5-min OBEO induction bath and does not indicate that OBEO was present during recovery. Left: continuous recovery recording; center: amplification of the final 5 s of recovery (295–300 s). Preceding treatments were 76 mg L⁻^1^ **A**, 85.5 mg L⁻^1^ **B**, 95 mg L⁻^1^ **C**, and 104.5 mg L⁻^1^ **D**. Recovery-period means are shown for QRS amplitude (mV) **E**, heart rate (bpm) **F**, RR interval (ms) **G**, PQ interval (ms) **H**, QRS duration (ms) **I**, and ST interval (ms) (**J**). ANOVA followed by Tukey’s test; data are mean ± standard deviation; *p < 0.05, **p < 0.01, and ***p < 0.001; *n* = 9

After transfer to anesthetic-free water, the ECG changes observed during OBEO exposure showed progressive reversal during the 5-min washout (Fig. 4A–D). Recovery was slower at higher preceding concentrations, and normalization was not complete for every ECG variable within the 5-min observation window. Therefore, these data demonstrate short-term movement toward baseline rather than complete cardiovascular recovery.

The recovery of cardiac activity in the group treated with 76 mg L⁻^1^ relative to the control group was 96.69% (Fig. 4A). Fish treated with 85.5 mg L⁻^1^ presented 95.41% heart rate recovery relative to the control group (Fig. 4B); the group treated with 95 mg L⁻^1^ of OBEO presented 93.64% (Fig. 4C), and the group treated with 104.5 mg L⁻^1^ presented 87.15% (Fig. 4D).

During recovery, the QRS complex amplitude for the control group presented a mean of 2.02 ± 0.17 mV, which was similar to the vehicle group (*p* = 0.999) and all groups treated during anesthesia recovery (*F* (5, 48) = 0.453, *p* = 0.808) (Fig. 4E).

During the recovery period, the control group presented a mean heart rate of 87.33 ± 7.28 bpm, which was like the vehicle group (p = 0.999) and those treated with 76 mg L⁻^1^ (*p* = 0.808), 85.5 mg L⁻^1^ (*p* = 0.516), and 95 mg L⁻^1^ (*p* = 0.173). However, it was higher than the group treated with 104.5 mg L⁻^1^ (76.11 ± 3.68 bpm). The group treated with 76 mg L⁻^1^ (84.44 ± 4.44 bpm) was similar to the groups treated with 85.5 mg L⁻^1^ (*p* = 0.996) and 95 mg L⁻^1^ (*p* = 0.854). The group treated with 95 mg L⁻^1^ (81.78 ± 4.52 bpm) was similar to the group treated with 104.5 mg L⁻^1^ (*p* = 0.157) (Fig. 4F).

The mean RR interval during recovery for the control group was 691 ± 56.76 ms, which was similar to the vehicle group (*p* = 0.944) and those treated with 76 mg L⁻^1^ (p = 0.900), 85.5 mg L⁻^1^ (*p* = 0.678), and 95 mg L⁻^1^ (*p *= 0.243); however, it was lower than the group treated with 95 mg L⁻^1^ (785.8 ± 47.42 ms). The group treated with 95 mg L⁻^1^ (735.3 ± 43.62 ms) was like the group treated with 104.5 mg L⁻^1^ (*p* = 0.133) (Fig. 4G).

The PQ interval during recovery in the control group was 115.9 ± 5.08 ms, which was similar to the vehicle group (*p* = 0.999), and groups treated with 76 mg L⁻^1^ (*p* = 0.891) and 85.5 mg L⁻^1^ (p = 0.488). The group treated with 76 mg L⁻^1^ (119.2 ± 3.42 ms) was like the 85.5 mg L⁻^1^ (p = 0.979) and 95 mg L⁻^1^ (*p* = 0.253) groups. The group treated with 85.5 mg L⁻^1^ (121.4 ± 5.41 ms) was like the group treated with 95 mg L⁻^1^ (p = 0.669) (Fig. 4H).

In the recovery period, the QRS complex duration for the control group (mean 20.22 ± 2.819 ms) was similar to the vehicle (p = 0.999) and groups treated with 76 mg L⁻^1^ (*p *= 0.996), 85.5 mg L⁻^1^ (*p* = 0.943), and 95 mg L⁻^1^ (*p* = 0.061); however, it was lower than the group treated with 104.5 mg L⁻^1^. The group treated with 76 mg L⁻^1^ (19.67 ± 2.06 ms) was similar to the group treated with 85.5 mg L⁻^1^ (*p* = 0.348). The group treated with 85.5 mg L⁻^1^ (22.00 ± 3.4 ms) was similar to the group treated with 95 mg L⁻^1^ (*p* = 0.759) (Fig. 4I).

In the recovery period, the ST interval for the control group was 290.7 ± 10.15 ms, which was similar to the vehicle group (*p* = 0.997) and groups treated with 76 mg L⁻^1^ (p = 0.460), 85.5 mg L⁻^1^ (p = 0.999), and 95 mg L⁻^1^ (*p* = 0.414); however, it was lower than the group treated with 104.5 mg L⁻^1^. The group treated with 76 mg L⁻^1^ (282.8 ± 6.96 ms) was similar to the group treated with 85.5 mg L⁻^1^ (*p* = 0.541). The group treated with 85.5 mg L⁻^1^ (290.1 ± 10.48 ms) was similar to the group treated with 95 mg L⁻^1^ (*p* = 0.341). The group treated with 104.5 mg L⁻^1^ (313.7 ± 10.70 ms) was higher than the other groups (Fig. 4J).

**Fig. 5:**
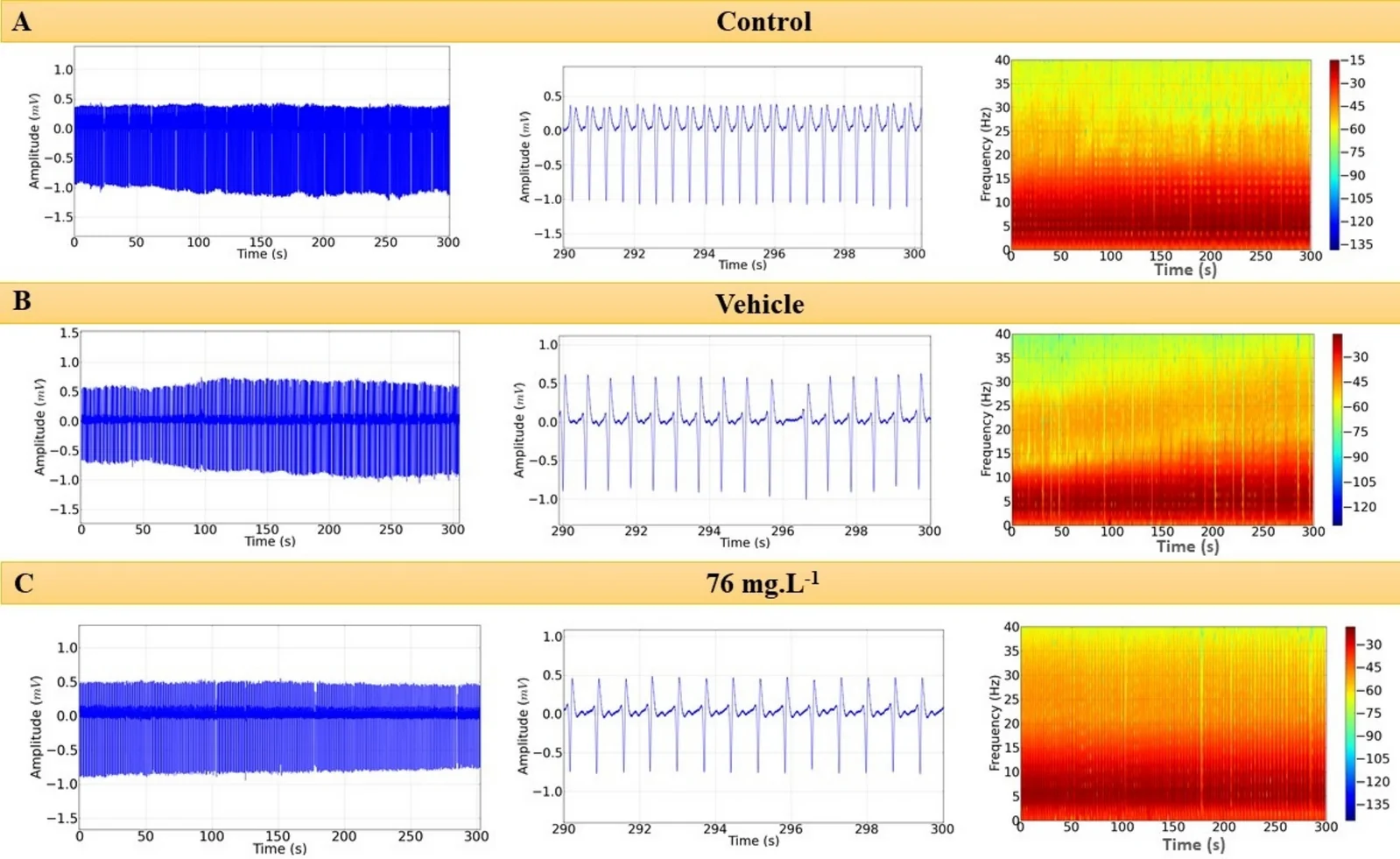

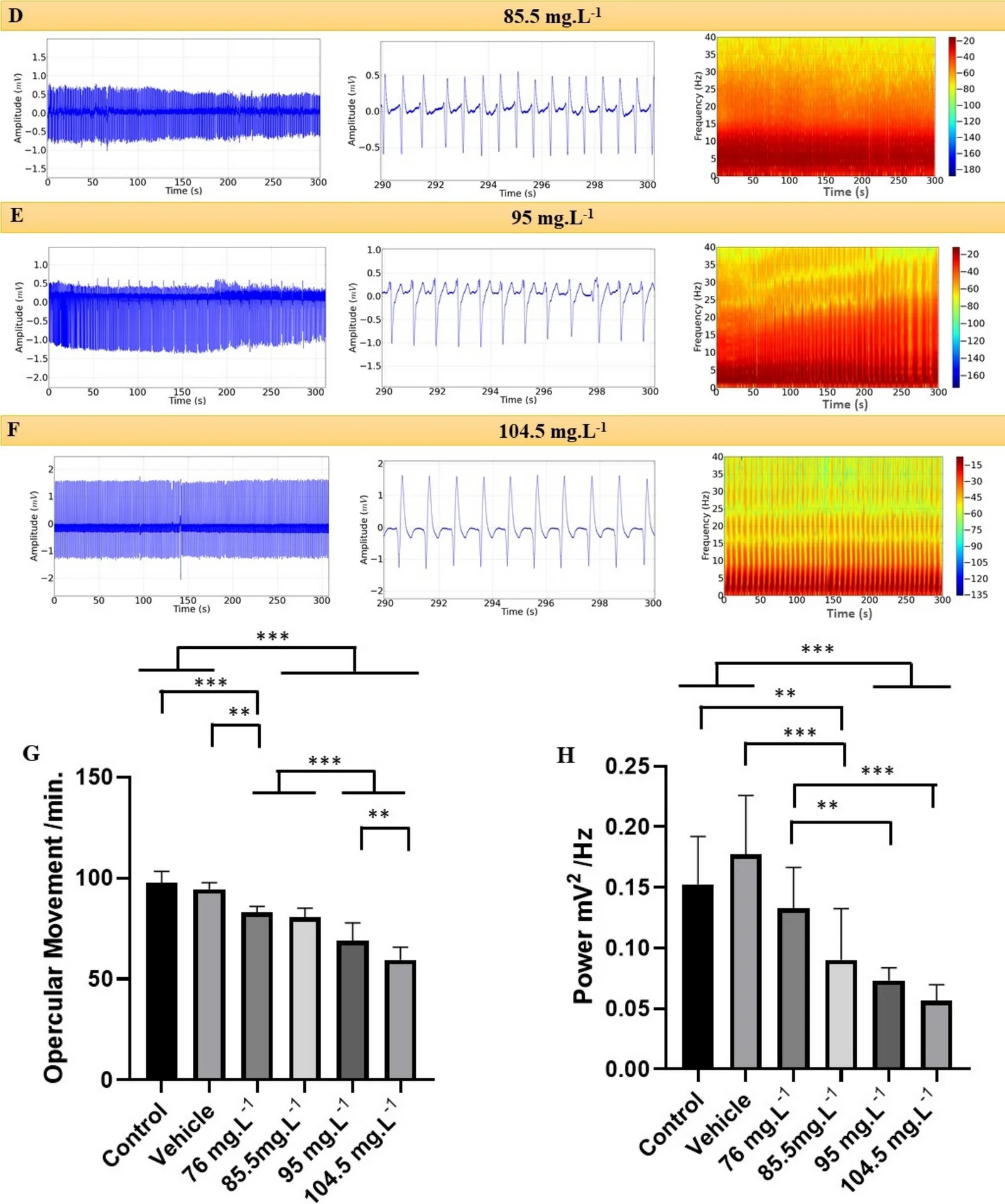
Recording of opercular activity in juvenile *Colossoma macropomum* during immersion bath at different OBEO concentrations (left). Amplification of the recording in the last 10 s (290–300 s) (center), recording of power distribution at the frequency of 40 Hz (right). Data obtained for the following groups: control group **A**; vehicle group **B**; group treated with 76 mg L⁻^1^ **C**; treated with 85.5 mg L⁻^1^ **D**; treated with 95 mg L⁻^1^ **E**; and treated with 104.5 mg L⁻^1^ **F**. Graph presenting the mean values of opercular movement per minute (OMM) **G** and mean opercular movement power values (mV^2^/Hz) **H**. (ANOVA followed by Tukey’s test; standard deviation. *p < 0.05, **p < 0.01, and ***p < 0.001; *n* = 9)

During treatment with OBEO at concentrations of 76 mg L⁻^1^, 85.5 mg L⁻^1^, 95 mg L⁻^1^, and 104.5 mg L⁻^1^, opercular movement frequency (OMM) showed a concentration-dependent reduction (Fig. 5A–F). The control group presented a mean of 97.67 ± 5.65 OMM, which was similar to the vehicle group (*p* = 0.823). However, they were higher than the treated groups. The group treated with 76 mg L⁻^1^ showed a 14.91% decrease in opercular beat relative to the control group. For the group treated with 85.5 mg L⁻^1^, it reduced by 17.41%; the group treated with 95 mg L⁻^1^ reduced by 29.36%; and the group treated with 104.5 mg L⁻^1^ presented a reduction of 39.15% (Fig. 5).

The group treated with 76 mg L⁻^1^ (83.11 ± 2.84 OMM) was similar to the group treated with 95 mg L⁻^1^ (*p* = 0.937), but they were higher than the other treated groups (Fig. 5G).

There was a reduction in power in the opercular movement (OMM) recordings during anesthetic induction. The control group presented a mean power of 0.152 ± 0.039 mV^2^/Hz, which was similar to the vehicle group (*p* = 0.674) and the group treated with 76 mg L⁻^1^ (*p* = 0.827). The group treated with 76 mg L⁻^1^ (0.132 ± 0.032 mV^2^/Hz) was similar to the group treated with 85.5 mg L⁻^1^ (*p* = 0.114). The groups treated with 85.5 mg L⁻^1^ (0.0901 ± 0.042 mV^2^/Hz), 95 mg L⁻^1^, and 104.5 mg L⁻^1^ were similar (*p* = 0.328) (Fig. 5H).

**Fig. 6:**
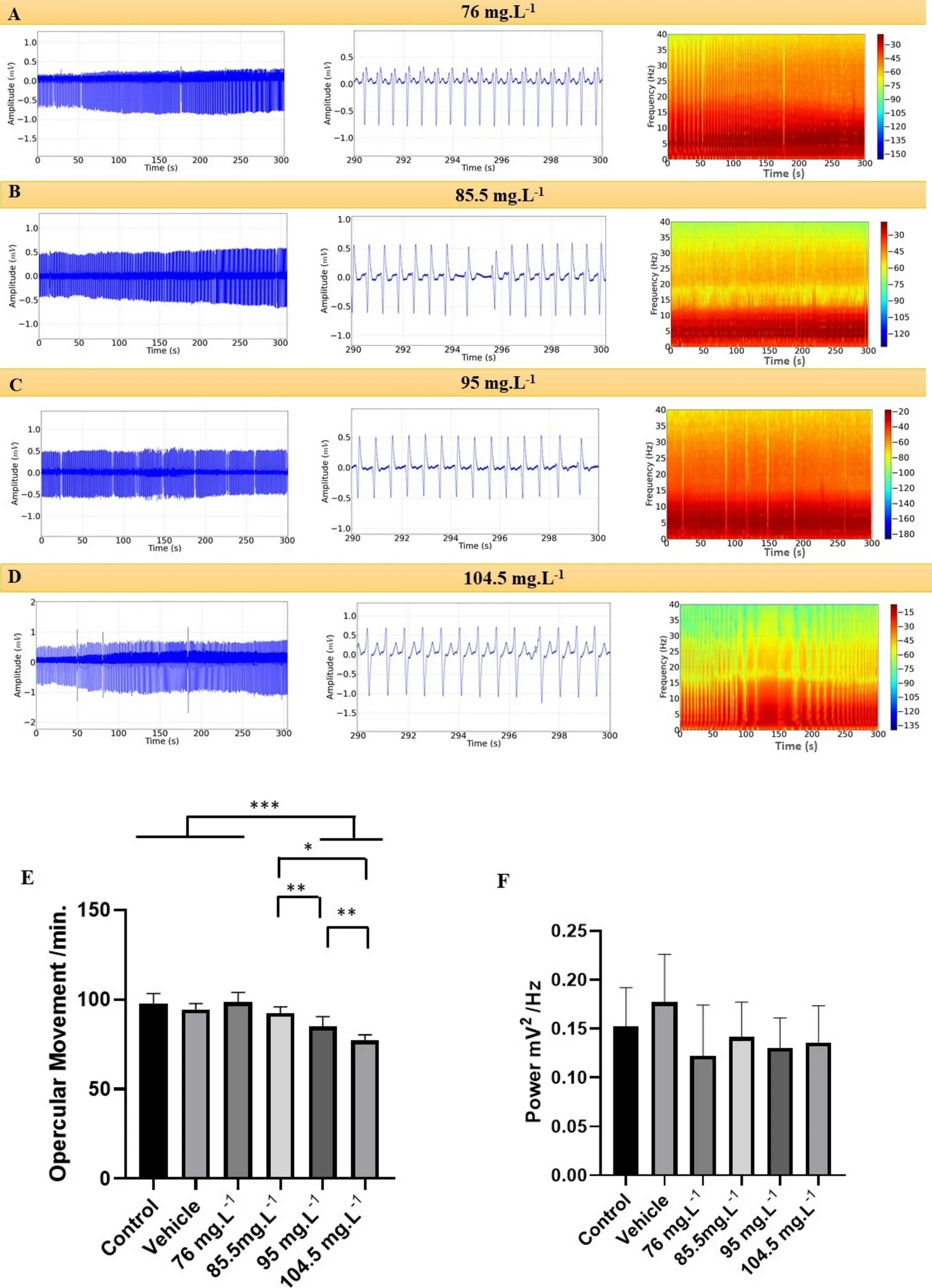
Recording of opercular beat during the recovery period (left), amplification of the recording in the last 10 s (290–300 s) (center), and spectrogram demonstrating power intensity up to 40 Hz during recovery after OBEO treatment (lasting 5 min) (right), evaluated in the following treatments: 76 mg L⁻^1^ **A**, 85.5 mg L⁻^1^ **B**, 95 mg L⁻^1^ **C**, and 104.5 mg L⁻^1^ **D**. Mean values of opercular movement per minute **E**; mean values of opercular movement power (mV^2^/Hz) **F**. (ANOVA followed by Tukey’s test; standard deviation. *p < 0.05, **p < 0.01, and ***p < 0.001; *n* = 9)

During the anesthesia recovery period, opercular movement frequency (OMM) showed pharmacological reversibility (Fig. 6A–D). The control group presented a mean of 97.67 ± 5.6 OMM, which was similar to the vehicle group (*p* = 0.662), the group treated with 76 mg L⁻^1^ (*p* = 0.997), and the group treated with 85.5 mg L⁻^1^ (*p* = 0.163). The group treated with 76 mg L⁻^1^ (98.67 ± 5.29 OMM) was similar to the group treated with 85.5 mg L⁻^1^ (*p* = 0.058) (Fig. 6E).

An increase in power in opercular movement (OMM) recordings was observed during anesthetic recovery. The control group presented a mean power of 0.1526 ± 0.039 mV^2^/Hz, which was similar to the other groups during recovery (*F *(5, 48) = 1.986; *p* = 0.097) (Fig. 6F).

## Discussion

The present study demonstrates concentration-dependent behavioral immobilization accompanied by cardiorespiratory and electrocardiographic modulation during short-term exposure to *Ocimum basilicum* essential oil. These results extend previous work on basil-derived preparations by integrating behavioral endpoints with direct cardiac and opercular recordings. Importantly, the behavioral definition used here was loss of postural reflex plus immobility. In fish anesthesia research, immobilization sufficient for handling should be distinguished from a surgical anesthetic plane, which requires additional assessment of responses to noxious stimulation and analgesic adequacy (Martins et al. 2019). Accordingly, the present data support the use of OBEO to produce a behavioral anesthetic/immobilization state for routine handling, but they do not establish surgical anesthesia.

The observed activity may be related to the antioxidant action of metabolites present in the extract, which can neutralize reactive oxygen species and reduce cellular damage. Previous studies have demonstrated that *O. basilicum* extracts rich in rosmarinic acid, eugenol, and linalool have protective effects in different experimental models, which is consistent with the results found in this study. On the other hand, toxicological studies indicate that estragole exhibits genotoxic and carcinogenic effects at high doses in animals (Pinheiro et al. 2017).

Basil oil showed an induction capacity relative to the applied dosage, with latency times becoming shorter, while the frequency of opercular movements and recovery times increased as the oil concentration increased to 76 mg L⁻^1^, 85.5 mg L⁻^1^, 95 mg L⁻^1^, and 104.5 mg L⁻^1^. Another study utilized increasing doses of OBEO, from 400 to 1000 µL L⁻^1^, and obtained the same characteristic for induction time, but did not present significant changes for the recovery period, despite the higher doses (Ventura et al. 2020).

Previous studies with OBEO in different fish species observed similar results. An ideal concentration for anesthesia of 300 mg L⁻^1^ was observed in a study (Yigit et al. 2022) with rainbow trout (*Oncorhynchus mykiss*) with a recovery time of 61 s; the increase between different concentrations, just as in the current study, resulted in shorter induction times and longer recovery times. Another study conducted with tambacus (a hybrid crosses between pacu, *Piaractus mesopotamicus*, and tambaqui, *C. macropomum*) demonstrated greater effectiveness for the concentration of 300 μL. L⁻^1^ of OBEO, with recovery times increasing with the concentration used, but induction times decreasing after this limit (de Araújo et al. 2023). The differences between these studies may result from variations between the chemical compounds used and between the animal species.

Cardiac activity appeared normal in the control group. The heart rate (HR) observed in this group (87.33 ± 7.28 bpm) differed slightly from mean values in other works involving anesthesia experiments in tambaquis. Similar to the findings of this study, Vilhena et al. (2022) observed a mean HR of 91 ± 9 bpm in juvenile tambaquis with a mass of 5.9 ± 1.5 g, and Barbas et al. (2017) reported an HR of 77 ± 6 bpm with mean mass values of 1.53 ± 0.39 g. These slight differences in HR may occur due to several factors, generally dependent on the environment and methodology, such as temperature, water quality, and techniques employed in ECG recording, as well as the mean mass values of the different groups (Souza et al. 2019).

The groups treated with OBEO presented HR reductions of 10.93%, 29.50%, 31.79%, and 40.83% for concentrations of 76, 85.5, 95, and 104.5 mg L⁻^1^, respectively, when compared to controls. Such a finding indicates that as the induced anesthetic concentration of OBEO increases the recorded mean HR values undergo reduction. Findings like these were found in the study by Vilhena et al. (2022) when analyzing the inverse relationship between increasing anesthetic concentration of *Piper divaricatum* essential oil (EOPD) and HR reduction in juvenile tambaquis during induction. Other studies that also analyzed the anesthetic effect of components present in natural products on cardiac function observed a decrease in HR in tambaquis (Souza et al. 2019; de Araújo et al. 2023).

QRS peak-to-peak amplitude did not differ significantly among groups, indicating that this specific voltage measure was preserved across the tested OBEO concentrations. By comparison, lidocaine at higher concentrations has been associated in tambaqui with bradycardia, QT prolongation, reduced QRS amplitude, and T-wave polarity inversion (Vieira et al. 2023). However, cross-agent comparisons should be interpreted cautiously because the present study did not quantify QT/QTc or HRV and did not directly assess cardiotoxicity. Therefore, preservation of QRS amplitude alone should not be interpreted as evidence that OBEO is safer than other anesthetics or as proof of cardiac safety.

During the 5-min washout, mean heart rate, ECG intervals, and respiratory variables generally moved toward control values, but recovery was slower at higher preceding concentrations and some variables remained different at 104.5 mg L⁻^1^. These findings support short-term reversibility of the measured mean responses, but they do not demonstrate complete restoration of cardiovascular autonomic regulation. Heart-rate variability (HRV) was not analyzed in this study. Because beat-to-beat HRV is influenced by autonomic regulation and can reveal anesthetic-specific cardiac effects that are not apparent from mean heart rate alone (Cotter and Rodnick 2006; Ling et al. 2022), the absence of HRV analysis and the 5-min recovery window are important limitations. Longer follow-up and time- and frequency-domain HRV analyses should be included in future studies.

Behavioral recovery was demonstrated by the progressive return of the postural reflex and spontaneous somatic activity in all treated groups, with longer latency at higher concentrations. This pattern is consistent with a dose-dependent anesthetic effect and indicates that the observed motor impairment was reversible within the behavioral observation period. However, behavioral recovery should not be equated with complete physiological recovery, because cardiovascular autonomic regulation may normalize on a different time scale (Ross and Ross 2008; Ling et al. 2022).

Opercular frequency and power decreased during induction and moved toward control values during washout, indicating that the ventilatory depression observed in this protocol was transient over the short observation period. No prolonged apnea or gross ventilatory failure was observed. Nevertheless, these short-term respiratory findings should be interpreted as evidence of reversibility under the tested conditions rather than as proof of a comprehensive safety profile.

The cardiorespiratory pattern observed with OBEO resembles transient responses reported for other plant-derived anesthetic agents in fish. Clove oil can produce dose-dependent bradycardia and respiratory depression with progressive recovery after anesthetic removal (Ross and Ross 2008). Likewise, essential oils and isolated monoterpenes have been associated with transient sinus bradycardia and changes in ECG intervals in fish (Barbas et al. 2016; Vilhena et al. 2022; de Araújo et al. 2023). International electrophysiological studies further show that different anesthetics can differentially affect QRS duration, QT interval, and HRV (Cotter and Rodnick 2006), reinforcing the need to characterize each candidate agent rather than infer cardiac safety from behavioral immobilization alone.

The use of *Ocimum basilicum* essential oil has demonstrated effectiveness in mitigating respiratory rate responses, since its application in appropriate concentrations attenuates secondary reactions to handling without causing deleterious effects on the energy metabolism of fish. This stabilization is evidenced by the maintenance of physio-metabolic homeostasis, avoiding glucose and lactate peaks that normally accompany handling without sedation (Ventura et al. 2020). The time until the onset of the anesthetic effect in tambaquis by basil essential oil varies according to the administered dosage (de Araújo et al. 2023), as can be observed in Fig. 2, which focuses on the loss of the postural reflex, one of the effects caused by anesthesia (Lima-Netto et al. 2016).

This pattern of increased or decreased time depending on the dose is repeated regarding opercular activity, as demonstrated by the study of Santos et al. (2026), which explores opercular frequency discrepancies with different administered amounts of OBEO (volumes of 10, 25, 50, 75, and 100 µL L⁻^1^), corroborating the results of Fig. 5A–F (volumes of 0, 76, 85.5, 95, and 104.5 mg L⁻^1^). The relationships described above demonstrate that increases in the proportions of OBEO to which tambaquis are exposed directly affect respiration rates (Fig. 5G) and opercular activity power (Fig. 5H).

*Ocimum basilicum* L. essential oil has been investigated as a potential alternative to anesthetics traditionally used in fish farming, such as MS-222, benzocaine, and eugenol, which may present limitations related to cost, availability, withdrawal requirements, or environmental considerations (Barbas et al. 2021; Santos et al. 2020; da Paz et al. 2024; de Souza et al. 2025). The present findings add short-term behavioral and cardiorespiratory information relevant to this potential application, but do not by themselves establish regulatory suitability or food-fish safety.

OBEO may offer practical advantages such as natural origin, availability, and potentially lower cost in some production settings (de Souza et al. 2024). However, these advantages should be considered separately from toxicological and regulatory safety. The current data identify OBEO as a candidate for further development as a short-duration immobilizing/anesthetic agent in tambaqui rather than as an already validated replacement for established aquaculture anesthetics.

Basil essential oil (*Ocimum basilicum*) has been shown to have a significant impact on the modulation of the GABA_A_ receptor, acting as an allosteric facilitator of the GABA (gamma-aminobutyric acid) system in the central nervous system. Since GABA is the main inhibitory neurotransmitter, responsible for promoting relaxation, reducing anxiety, and inducing sedation (Böhm et al. 2025) our results corroborate the action of OBEO as a central nervous system depressant that can induce anesthesia in fish.

Furthermore, constituents present in OBEO have been reported to modulate sodium, calcium, and potassium channels and to interact with TRPV1-related pathways, mechanisms that could plausibly contribute to the cardiac and respiratory effects observed during exposure (Vatanparast et al. 2017; Pinheiro et al. 2017; Rivera-Ruedas et al. 2025). However, the present study did not directly assess ion-channel function, ectopic activity, arrhythmia induction, or antiarrhythmic efficacy. Therefore, the ECG changes observed here are compatible with electrophysiological modulation but do not demonstrate antiarrhythmic activity of OBEO.

In this sense, to leverage the potential of OBEO as an anesthetic in aquaculture, it is valid to point out that there is wide variation in the composition of this essential oil depending on the origin of the plant from which the product to be used was extracted; thus, the main compound of the chromatography, a terpenoid or phenylpropanoid responsible for the anesthetic action, differs according to the *Ocimum basilicum* individual from which the oil was extracted (Telci et al. 2006). An example of this is the chromatographic analysis of the OBEO used in the present study, which presents Estragole (methyl chavicol) as its main compound. Analogously, the OBEO used in the study by Ventura et al. (2020) also exhibited estragole as the main compound.

The OBEO used here represents an estragole-rich chemotype, and this chemical profile is important when considering translation beyond the acute experimental setting. Estragole can undergo hepatic bioactivation to reactive metabolites, and toxicological concerns have been reported under high-dose or chronic exposure conditions. The present experiment evaluated only short-term physiological responses and cannot address systemic toxicology, pharmacokinetics, metabolism, tissue residues, or consumer exposure. Consequently, food-fish application would require dedicated studies of these endpoints and establishment of an appropriate withdrawal period before regulatory or food-safety conclusions can be made.

Additional limitations should be considered. First, anesthesia depth was defined behaviorally by loss of posture and immobility, without testing responses to noxious stimulation; therefore, a surgical plane was not demonstrated. Second, the 5-min recovery window and normalization of mean values cannot establish complete physiological recovery, and HRV was not analyzed. Third, QT/QTc could not be measured reliably because the terminal T-wave boundary was not consistently identifiable, limiting assessment of ventricular repolarization. Finally, the study did not evaluate pharmacokinetics, tissue residues, chronic toxicity, or withdrawal time. These limitations constrain the conclusions to short-term behavioral and cardiorespiratory effects under the specific experimental conditions used.

## Conclusion

*Ocimum basilicum* essential oil induced a concentration-dependent behavioral anesthetic/immobilization state in juvenile tambaqui (*Colossoma macropomum*), characterized by loss of postural reflex and immobility. Higher concentrations shortened induction latency but prolonged behavioral recovery. Because responses to noxious stimuli were not evaluated, the results support immobilization for routine short-duration handling and do not establish a surgical plane of anesthesia.

Electrocardiographic and opercular recordings demonstrated concentration-dependent sinus bradycardia, prolongation of selected ECG intervals, and ventilatory depression during OBEO exposure. These responses generally moved toward baseline during a 5-min washout, although recovery was slower and not complete for all ECG variables at the highest concentration. No arrhythmias or persistent gross physiological abnormalities were observed during the short experimental period, but unchanged QRS amplitude alone cannot establish cardiac safety, and QT/QTc and HRV were not assessed. Thus, OBEO should be regarded as a promising candidate for further evaluation rather than a definitively safe anesthetic for food-fish production. Studies addressing longer-term cardiovascular recovery, nociceptive endpoints, pharmacokinetics, estragole toxicology, tissue residues, and withdrawal periods are required before broader aquaculture and food-safety recommendations can be made.

The study presents limitations regarding the assessment of stress induced by animal handling, such as measurements of cortisol, lipid peroxidation, and glutathione peroxidase.

## Data Availability

All data generated or analyzed during this study are available in the public repository at the following link: https://drive.google.com/file/d/1U88cOikf-L6JeTorXowwnA2ilQSWmk65/view?usp=sharing. The datasets support the findings of this study and include the original electrophysiological and behavioral data
